# An automated solid waste detection using the optimized YOLO model for riverine management

**DOI:** 10.3389/fpubh.2022.907280

**Published:** 2022-08-12

**Authors:** Nur Athirah Zailan, Muhammad Mokhzaini Azizan, Khairunnisa Hasikin, Anis Salwa Mohd Khairuddin, Uswah Khairuddin

**Affiliations:** ^1^Department of Electrical Engineering, Faculty of Engineering, Universiti Malaya, Kuala Lumpur, Malaysia; ^2^Department of Electrical and Electronic Engineering, Faculty of Engineering and Built Environment, Universiti Sains Islam Malaysia (USIM), Negeri Sembilan, Malaysia; ^3^Department of Biomedical Engineering, Faculty of Engineering, Universiti Malaya, Kuala Lumpur, Malaysia; ^4^Centre of Intelligent Systems for Emerging Technology (CISET), Faculty of Engineering, Universiti Malaya, Kuala Lumpur, Malaysia; ^5^Malaysia Japan International Institute of Technology, Universiti Teknologi Malaysia, Kuala Lumpur, Malaysia

**Keywords:** computer vision, image processing, object detection, smart city, urbanization, water quality

## Abstract

Due to urbanization, solid waste pollution is an increasing concern for rivers, possibly threatening human health, ecological integrity, and ecosystem services. Riverine management in urban landscapes requires best management practices since the river is a vital component in urban ecological civilization, and it is very imperative to synchronize the connection between urban development and river protection. Thus, the implementation of proper and innovative measures is vital to control garbage pollution in the rivers. A robot that cleans the waste autonomously can be a good solution to manage river pollution efficiently. Identifying and obtaining precise positions of garbage are the most crucial parts of the visual system for a cleaning robot. Computer vision has paved a way for computers to understand and interpret the surrounding objects. The development of an accurate computer vision system is a vital step toward a robotic platform since this is the front-end observation system before consequent manipulation and grasping systems. The scope of this work is to acquire visual information about floating garbage on the river, which is vital in building a robotic platform for river cleaning robots. In this paper, an automated detection system based on the improved You Only Look Once (YOLO) model is developed to detect floating garbage under various conditions, such as fluctuating illumination, complex background, and occlusion. The proposed object detection model has been shown to promote rapid convergence which improves the training time duration. In addition, the proposed object detection model has been shown to improve detection accuracy by strengthening the non-linear feature extraction process. The results showed that the proposed model achieved a mean average precision (mAP) value of 89%. Hence, the proposed model is considered feasible for identifying five classes of garbage, such as plastic bottles, aluminum cans, plastic bags, styrofoam, and plastic containers.

## Introduction

The production of solid waste is one of the main concerns in urban clusters. Urbanization and population growth have been identified as major indicators of the growing rate of solid waste, particularly in water bodies, such as rivers, lakes, seas, and oceans (1). River ecosystems are the main elements in the universal water cycle and are vital for human health since rivers connect the inland watersheds to the marine environment (2). Hence, rivers act as channels that link terrestrial and aquatic realms. Nevertheless, pollutants may accumulate from various situations in the waterways (3). For example, microplastic pollution in aquatic systems is a progressively prevailing international problem that contributes to severe impacts on ecosystem functioning and human health (3, 4). Generally, urbanization is the main factor in the increment of microplastic loads along a subtropical river system. The interference of plastic in the food chain is a huge concern for human health. This is because when plastic is exposed to natural forces, plastic breaks down into microplastic and nanoplastic particles, which contain chemical components that can enter the tissues of marine organisms, including species consumed by humans. Research on associations among micro- and nanoplastic exposure, toxicology, and human health are actively performed in previous works. It can be seen that the impacts of pollution affect the ecological system and humans as well (5–7).

Managing solid waste appropriately in the riverine ecosystem is important for building sustainable cities, yet it remains a challenge for certain developing countries and cities. In the absence of automation and modernization of waste management operations, the collection of solid waste remains to be labor-intensive activity. Hence, this work proposed an automatic solid waste collecting system. Several initiatives have been adopted to manage pollution, for example, manual and machine-based cleaning require human supervision constantly. In addition, the requirement of manual labor for cleaning waste can be a threat to the person (8–10). Hence, an autonomous cleaning robot that can clean waste from the water contributes to a significant impact on river pollution control. However, the suitable design of the robot is a challenging task. Riverine monitoring has become an important first step for most countries and an automated system has been demanded to support their efforts (11, 12). Research studies on designing cleaning robots are increasingly becoming ubiquitous. Cleaner robots can be applied to lessen the labor volume of sanitation workers and improve the land and water ecological environment (13–16). The main tasks to be performed by cleaner robots are garbage detection and garbage collection. Garbage detection is mainly important since it is responsible for providing accurate object location information for the cleaning robot. Accurate garbage detection will aid the garbage collection task to be performed efficiently. Therefore, an efficient object detection method that incorporates computer vision is highly demanded.

Generally, computer vision is an area of artificial intelligence (AI) that permits computers and systems to interpret information from various visual inputs. Computer vision aims to replicate the capability of human vision for computers or devices to understand images. Object detection is a computer vision method that aims to detect the location of target objects in images or videos. The detection process includes two parts: (1) the category information and probability of the target and (2) the location of the target by applying bounding boxes with labels (17–23). Recent developments in computer vision methods have achieved significant improvements in various applications, such as in medical diagnosis (17, 18), object detection (19, 20), precision agriculture (21), transportation system (22), and biometric system (23). With the expansion of deep learning algorithms in machine vision applications, deep learning techniques have attained state-of-the-art outcomes for the object detection system. Besides that, the deep learning method also has the capability to automatically extract deep features from the input image by adopting self-learning (24–26). This is possible since deep learning permits computational models to learn and signify data with several levels of abstraction impersonating how the brain distinguishes multimodal information. Hence, this work focuses on developing a novel object detection model by incorporating several improvements to the conventional YOLOv4 model architecture. The contributions of this paper are as below:

- A novel object detection algorithm that automatically detects garbage by using a computer vision approach.
- The proposed object detection model promotes rapid convergence that improves the training time duration.
- The proposed object detection model improves the detection accuracy by strengthening the non-linear feature extraction process.

This paper is arranged as follows. Section Background study discusses the background study of object detection methods. Next, Section Proposed method explains the methodology of the proposed method. Experimental results are tabulated and discussed in Section Results and discussion. Lastly, Section Conclusion concludes the paper.

## Background study

The mainstream object detection algorithms are based on convolution neural networks (CNN), which are one-stage and two-stage detections, using different feature extraction methods. Object detection algorithms that adopt a two-stage detection method include regions with CNN (R-CNN), fast regions with CNN (Fast R)-CNN, and Faster R-CNN, which divide the detection task into (1) region proposal and (2) classification. Meanwhile, the one-stage detection method integrates region proposal and classification into one step, which reduces the detection time. The mainstream methods of one-stage detection are Single Shot Detector (SSD) and YOLO (27).

In recent years, deep learning algorithm in computer vision is a rapidly developing research topic in classifying floating debris. The work in Fulton et al. (28) classified three classes of trash, which are plastic debris, biological materials, and man-made objects. The work applied the Faster R-CNN method and resulted in 81 mAP of performance when classifying 820 test images. The trash detection system proposed in Fulton et al. (28) applied the two-stage detection approach. Region proposal network (RPN), which is a fully convolutional network (FCN), predicts object boundaries and confidence scores at each point of location simultaneously. The RPN is aimed to produce high-quality region proposals. RPN is combined with Fast R-CNN to form a network by sharing their convolutional features to produce Faster R-CNN. In Faster R-CNN, regions of interest are generated from the input image and these are transferred to subsequent convolutional layers. The RPN generates region proposals using the previously generated feature map. RPN adopted a sliding window over the feature maps while each window will generate k anchor boxes of different shapes and sizes. Then, adjacent pixels are clustered by texture, color, or intensity into the classifiers. After training, the classifiers on each region proposal will be returned for object detection purposes (29, 30). Despite the high localization and recognition accuracy, a two-stage detection approach suffers from slow detection speed and is not applicable for real-time applications.

On the other hand, a one-stage detection approach based on the YOLOv3 model is applied in the garbage detection system in Watanabe et al. (12) and Li et al. (16). The work in Watanabe et al. (12) demonstrated YOLOv3-based object detection for monitoring marine debris with an mAP of 77.2% by using 37 test images. Meanwhile, the work in Li et al. (16) developed a vision-based water surface garbage capture robot using a modified YOLOv3 model that is able to detect plastic bottles, plastic bags, and styrofoam. The performance of the model was evaluated based on 301 test images and achieved an mAP of 91.4%. The works in Watanabe et al. (12) and Li et al. (16) solved object detection as a regression problem that considers the whole image as input and instantaneously produces class probabilities and multiple bounding boxes. Hence, this has made the detection model much faster compared to the two stages of object detectors. A You Only Look Once (YOLO) detector adopts the whole image as the network input, which will then be separated into an s × s grid. Then, the model will provide the position of the object border and the corresponding class in the output layer (31). The idea of the YOLO detector was to employ an exclusive neural network to the whole image, whereby the network splits the image into sections and concurrently predicts probabilities and bounding boxes for each section. The weight of the bounding boxes is computed based on the predicted probabilities. Each bounding box will have its confidence score and the prediction is produced as a static number of boundary boxes. The prediction detects one object for each grid cell by applying a non-maxima suppression algorithm. YOLO usually adopts ImageNet to pre-train parameters and then applies a target detection dataset to recognize the training (32). Nevertheless, previous works in Refs. (12, 16, 28) focused on a small number of test images, which is <1,000 test images. As more test images are considered, the classification process will become more complex. Hence, a more effective object detection algorithm is required to achieve good classification accuracy.

Based on previous works reported in Junos et al. (27); Morera et al. (32), SSD and YOLO detectors have provided feasible outcomes under various conditions with respect to image sizes, illumination, viewing perspectives, incomplete occlusion, and complex background. The benefit of applying the SSD model was the exclusion of False Positive (FP) cases. On the contrary, YOLO had shown to give better object localization results detecting a higher number of True Positive (TP) panels with higher accuracy. Moreover, YOLOv4 had shown to have significantly good precision and a real-time object detection algorithm that combined the features of YOLOv1, YOLOv2, and YOLOv3. In addition, YOLOv4 can achieve the existing optimum detection speed with a trade-off in detection accuracy. On the other hand, YOLOv3-tiny and YOLOv4-tiny are the lightest versions of the YOLOv3 model and YOLOv4 model, respectively. Although the YOLO-tiny models are simpler and less complex structures, the detection performance of the YOLO-tiny models was reduced significantly due to the weak feature extraction process (27).

Generally, the model structure of YOLOv4 consists of backbone, neck, and prediction. By adopting the learning ability of Cross-Stage Partial Network (CSPNet), YOLOv4 built the CSPDarkNet53. Darknet-53 is a convolutional neural network that consists of 53 layers deep and functions as a backbone of the YOLOv4 model. Meanwhile, the Neck includes the Spatial Pyramid Pooling Network (SPPNet) and Path Aggregation Network (PANet). SPPNet focuses on removing the fixed-size constraint of the network. In SPPNet, the feature layer is convolved three times, followed by the input feature layer, which is maximally pooled by applying the maximum pooling cores using different sizes. Then, PANet convolves the feature layers after the operation of Backbone and SPPNet. PANet enhances the segmentation process by conserving spatial information. This is computed by accurately localizing the signals in lower layers and adopting path augmentation, which lessens the information path between lower layers and the topmost feature. Moreover, YOLOv4 adopted a new mosaic data augmentation technique with the aim to increase the dataset and presented DIOU as the positioning loss function (25). Hence, the network tends to optimize toward increment of overlapping areas, therefore successfully improving the accuracy. With the increasing number of layers in the convolutional neural networks, the depth of the network deepens, and the deeper network structure is useful for the feature extraction process. On the other hand, the prediction module performs predictions based on the features obtained from the network. The prediction results will tune the positions of the three preceding frames, and lastly, they will be filtered by the non-maximum suppression (NMS) algorithm to attain the final prediction frame (33–36). In a complex situation that includes external interference, such as occlusion and multi-scale, there are still some limitations in the garbage detection when applying the conventional YOLOv4 model, such as long training time, high computation cost, and overfull parameters. Besides, the conventional YOLOv4 model also suffers from insufficient shallow feature extraction for the multi-scale object. Therefore, this work focuses on improving the conventional YOLOv4 model architecture in detecting the floating debris for the river monitoring system. The improvements performed in the proposed YOLO model include (i) modification of CSPDark-Net53 into the backbone to overcome limitations due to training time, (ii) adoption of the Hard-Swish activation function, and (iii) improved PANet in the Neck module to aid the feature extraction process.

## Proposed method

### Image dataset and transfer learning

In this work, the training images are obtained under various conditions in terms of brightness and positions to prevent overfitting (33). Five classes of the floating debris database are developed, which include styrofoam, plastic bags, plastic bottle, plastic containers, and aluminum cans (Table 1). Pre-trained convolutional weights are applied for the training process to improve the accuracy of the object detector and lessen the computation time. Generally, applying inadequate learning data will produce inaccurate object detection performance. Thus, transfer learning is applied to aid the training process and obtain substantial results without having to include massive data (37). Hence, this work adopted transfer learning and applied the pre-trained weights from Microsoft Common Objects in Context (MS-COCO) dataset to improve the model performance. The weights of the convolutional layers for the proposed model are pre-trained based on the MS-COCO dataset. The MS-COCO dataset is a large-scale image dataset that contains annotations that enable users to train the computer vision models to recognize, label, and describe objects. In addition, the MS-COCO dataset complements the transfer learning process where the data is applied for one model which serves as an initial point for another. The MS-COCO dataset is an important benchmark for computer vision to train, test, and refine the object detection model. The formerly learned MS-COCO features provide the model with additional image recognition necessities required for the object detection process (38).

**Table 1:** The training and test datasets.

**Object class**	**Training images**	**Test images**
Plastic bottle	3,798	1,085
Aluminum can	2,799	586
Plastic bag	2,060	551
Styrofoam	487	146
Plastic container	410	113
Total	9,554	2,481

### The proposed optimized YOLO model

This work proposed a novel detection model for the automatic garbage detection system that consists of three stages: backbone feature extraction network, neck network, and object detection stage. The novelty of the proposed work is the development of an improved YOLO model by optimizing the network structure of a YOLOv4 model to detect solid wastes from the image dataset. Several improvements are performed to optimize the network structure of the conventional YOLOv4 model, such as follows: (1) CSPDarkNet53 is improved by adopting CSP1_X with the aim to reduce network modules which will lead to a reduction of parameters in feature extraction, (2) Hard-Swish (H-Swish) activation function is adopted to strengthen the non-linear feature extraction ability of the network, and (3) PANet in the Neck module is improved by adopting CSP2_X to enhance feature extraction and enhance the accuracy of the model. Finally, the performance of the proposed algorithm is compared with previous works to justify the contribution of the proposed model.

#### Backbone feature extraction network

**Figure 1:**
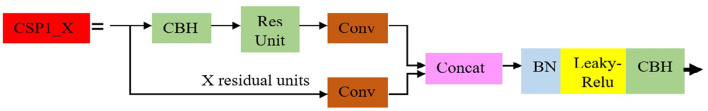
The fine-tuned module structure.

A residual module is proposed for the conventional YOLO-v4 to improve the network's learning ability and reduce the parameters of the network. The computation of the residual module (Res-unit) can be summarized as follows. First, a 1 × 1 convolution process is performed followed by a 3 × 3 convolution; then, the two outputs of the module are weighted. The weighting computation is performed to increase the information of the feature layer without altering the dimension data. In CSPDarkNet53, the input is the set of feature layers of the image, and then down-sampling convolution is executed continually to obtain better semantic information. Consequently, the last three layers of Backbone have the highest semantic information, and then the last three layers of features are selected as the input of the Spatial Pyramid Pooling Network (SPPNet) and Path Aggregation Network (PANet). Though YOLOv4 applies a residual network to decrease the computing power requirement, its memory requirement still needs to be improved. Therefore, in this work, the network structure of CSPDarkNet53 of YOLO-v4 is modified to the CSP1_X, as shown in Figure 1.

Hard-Swish (H-Swish) activation function plays an important role in weight reduction. As compared to CSPDarkNet53, the modified network adopts the H-swish activation function (34), as presented in Equation (1):


(1) $$H-swish\left(x\right)=x\frac{ICIC\;(x+3)}{6}$$

In this case, the Swish function includes the Sigmoid function, which leads to a higher computation cost compared to the ReLU function. However, the Swish function has been shown to be more effective than the ReLU function (34, 35). The work in Ref. (35) also presented the capability of the H-swish function on reducing the number of entries in the model's memory. Hence, this work applied the H-Swish function to further reduce the computation time and justify the benefits of the H-swish function in reducing the computation time and concurrently ensuring no gradient explosion and disappearance.

In the fine-tuned module structure as shown in Figure 1, the input feature layer of the residual block is separated into two parts. The first part is used as the residual edge to perform the convolution operation. The second part performs the role of the trunk which computes 1 × 1 convolution at first, then performs 1 × 1 convolution to fine-tune the channel after incoming the residual block. Accordingly, it performs the 3 × 3 convolution process to improve the feature extraction. Finally, the two parts are concatenated, therefore integrating the channels to gain more feature layer information. In this work, three CSP1_X modules are adopted in the improved Backbone, where X denotes the number of residual weighting operations in the residual structure. Consecutively, after stacking, a 1 × 1 convolution is computed to integrate the channels.

#### Neck network

**Figure 2:**
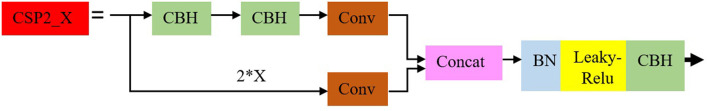
CSP2_X module structure.

The neck network consists of SPPNet and the modified PANet. In this work, the SPPNet component expands the acceptance range of backbone features efficiently, and this contributed to the separation of the vital contextual features. The high computational cost of model reasoning is mostly produced by recurrent occurrences of gradient data in the network optimization process. Therefore, this paper proposes the CSP2_X module into PANet to split the basic feature layer from Backbone into two sections and then reduce the repeated gradient information *via* a cross-stage process. The CSP2_X model architecture is presented in Figure 2.

The adoption of an improved CSPNet network module will have the ability to improve the network feature fusion stage. The collective process can perform the top to down transmission using deeper semantic features in PANet. Simultaneously, the bottom to up deep features is fused from the SPPNet network, hence appreciating feature combination between different backbone layers and different detection layers in the Neck network to provide discriminant features for the prediction network.

#### Object detection stage

The proposed detection model commences with understanding an image by applying logical *SxS* grids and computing the weighted feature sets to obtain a probability on each area of cells. If the middle of a possible object belongs to one of the cells, a preliminary bounding box will be created based on the prediction probability as determined by the trained model in (2) (36).


(2) $$\mathrm{Pr}\left(object\right)=\{\begin{array}{ll} 0, & has\;potential\;objects\\ 1, & has\;no\;potential\;objects\; \end{array}$$

Then, the detection model performs the prediction process using *K* numerous boxes and extracts a 3D tensor based on (3), where C denotes the defined number of classes.


(3) S∗S∗(K∗(4+1+C))

**Figure 3:**
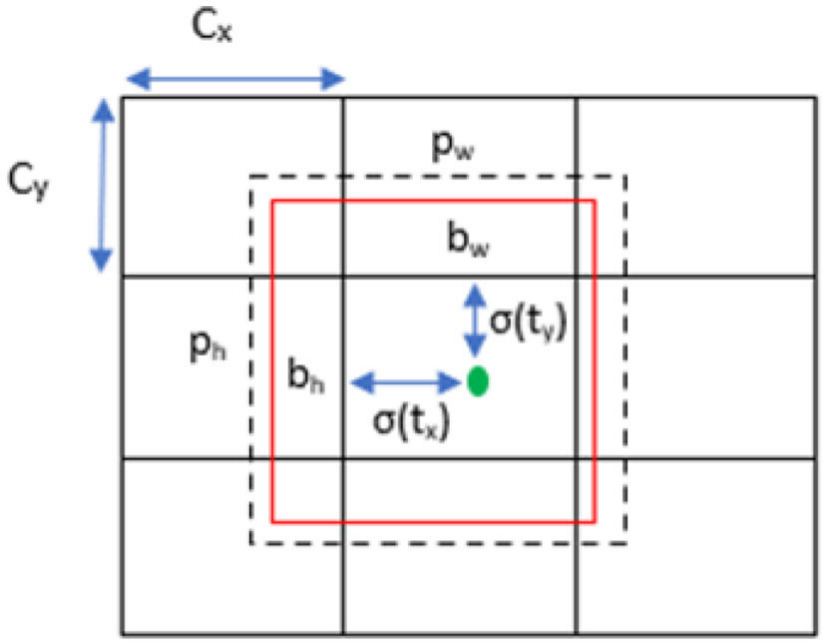
Bounding box prediction with specifications.

In Figure 3, the prediction's bounding box is computed based on the width pw and height ph, which had offsets cx and cy from the cluster centroid. Once the cell is offset from the upper left by (cx, cy) and the box takes values of pw and ph, then the prediction is computed as (4–7).


(4) $$b_{x}\;=\;\sigma \left(tx\right)+\;cx\;$$

(5) $$b_{x}\;=\;\sigma \left(tx\right)+\;cx$$

(6) $$b_{w}\;=\;p_{w}e^{t_{h}}$$

(7) $$b_{h}\;=\;p_{h}e^{t_{h}}$$

During the formation of bounding boxes, the intersection over union (IoU) computes the matching of the prediction with the ground truth image. The confidence score decreases if the originally predicted object does not represent the ground truth, hence, resulting in an unsatisfied prediction. For each cell, C is computed based on Pr (Classi|Object). As shown in (8), the object that fulfills the threshold will obtain an initial bounding box despite having multiple predictions over the specific object.


(8) $$Pr(Class_{i}|Object)*Pr(Object)*IoU\;=\;Pr(Class_{i})*IoU$$

This work adopted Bag of Specials (BoS), cross-stage partial connections (CSP), and multi-input weighted residual connections (MiWRC). BoS is simply defined as a set of modules that have a significant improvement on the accuracy of the object detection with a small increment of inference cost. Meanwhile, the BoS features for the proposed YOLOv4 detector in this work also included distance-IoU-non-maximum suppression (DIoU-NMS). DIoU is applied by computing the IoU and the distance among the central points of two bounding boxes during the suppression of redundant boxes. The rest of the boundary boxes that predict a similar object will be filtered by NMS while maintaining the one with the highest confidence value. This increases its robustness when it comes to cases with occlusions.

## Results and discussion

**Figure 4:**
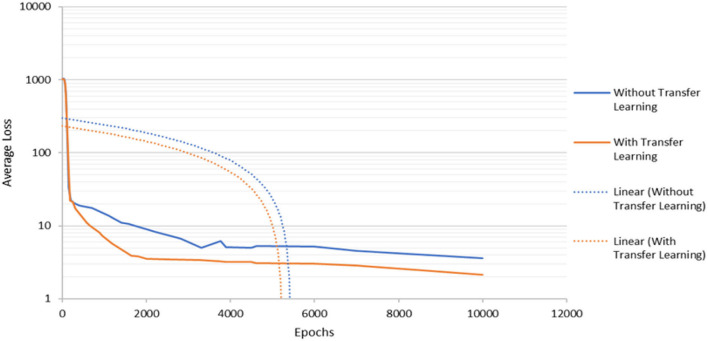
The detection model's convergence rate during the training process.

The experiments in this work are executed using an epoch of 10,000 and the input image size of 416 × 416 × 3. First, this study investigates the contribution of transfer learning to the performance of the object detector by applying the MS-COCO dataset, in which the pre-trained convolutional weight is applied in the training stage. Figure 4 shows that the detection model with transfer learning provides a better converging rate compared to the model without transfer learning. The model that applied transfer learning continues to decrease its average loss until it achieves training steps of approximately 1,800 where the average loss converges to a constant level. This event shows a small value of decrement in the average loss until the termination of the training process. In the meantime, the model without transfer learning suffered from overfitting at about 3,700 training. This means that the detection model does not generalize well without including transfer learning. As a result, the overfitted model will not be able to perform well on the new test dataset which will affect the accuracy of the detection system. After some time, the average loss for both models constantly decreases but the model without transfer learning has a higher average loss until the end of the training process. Hence, this shows that the application of the transfer learning process will contribute to lower generalization error during the training process which will aid the detection process on the new test dataset.

**Figure 5:**
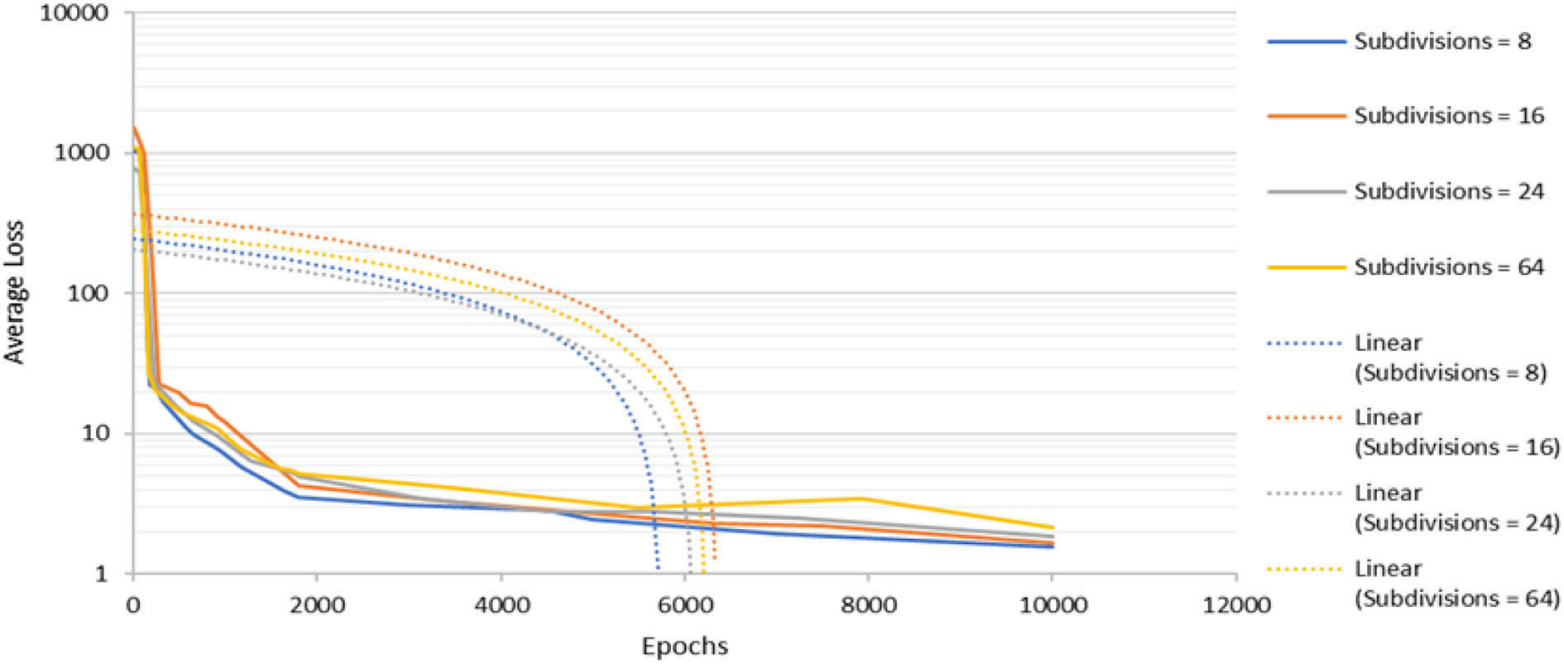
The detection model's convergence rate for various subdivisions parameter.

To determine the optimized network structure of the proposed model, the subdivision parameter is varied between 8 and 64 while keeping other parameters constant. Subdivision simply means the batch is split by the value of subdivision into mini batches. For example, for batch=64 and subdivision=8, the training will have 64/8 = 8 images per mini batches. These mini batches will then be sent to the GPU for the computation process. The computation process will be repeated 8 times until a batch is completed. The new iteration begins to be 8 in this work because it produces the lowest average loss during the training process. Averaging over more images helps to speed up the time for training and to generalize the training even more. However, this can be a problem when the subdivision is reduced because a memory issue might occur if the GPU does not have enough memory to process more images at one time. From Figure 5, it can be seen that when the subdivision is set to 64, the average loss drops drastically between 0 and 1,800 epochs with irregular fluctuations before it becomes overfit at about 8,000 epochs. As for subdivisions parameters 16 and 24, the pattern for both is quite similar. It can be observed that the lower the subdivisions parameter, the lower the average loss is at the end of the training. The model with subdivision = 8 has the lowest value in terms of average loss throughout the training. Hence, the subdivision parameter is chosen for weights. Besides that, the proposed work required the shortest time to train the image dataset as compared to other models.

Moreover, various pre-trained convolutional weights as feature extraction mechanisms are compared. This analysis was performed to determine the optimized convolutional weights for the network. In addition, the loss function of the proposed model is optimized by computing the IoU thresholds, which indicate the degree of overlap area between the target bounding box generated by the proposed model and the original labeled bounding box. In this work, the IoU loss function is optimized by applying the DIoU-NMS. In the training process, the adoption of DIoU loss in the network loss function has been shown to decrease the number of iterations and improve the degree of the overlap area. Hence, this contributes to higher detection accuracy. Several experiments have been performed to verify the effectiveness of the optimization approach. The evaluation parameters used in this work are precision, recall, mAP, and F1 score. Based on Table 2, the proposed work produced the highest mAP, F1 score, and recall values compared to other convolutional weights. In summary, Table 3 tabulates the optimized hyperparameters of the proposed model. The proposed model continuously optimizes the parameters throughout the training process with the aim to speed up the network convergence and avoid overfitting.

**Table 2:** The detection performance for different convolutional weights.

**Convolutional**	**mAP**	**F1-Score**	**Recall**	**Training**
**weights**	**(%)**		**(%)**	**time (hours)**
Yolov4-csp.conv.142	58.5	0.6	55	10
Yolov4-sam-mish.conv.105	70.9	0.7	64	10.3
Cspx-p7-mish_hp.344.conv	55.2	0.6	51	12.5
Darknet19_448. conv. 23	50.2	0.5	47	12.5
Yolov4. conv. 137	71.2	0.7	68	11
The proposed work	89.0	0.8	86	7.5

**Table 3:** The hyperparameters of the proposed model.

**Hyperparameters**	**Values**
Initial learning rate	0.00100
Epoch	10,000
Batch size	64
Subdivisions	8
Optimizer weight decay	0.00050
Momentum	0.84900
Classification coefficient	0.20600
Hue	0.01700
Saturation	1.50000
Exposure	1.50000
Value	0.50000
Scale	0.10000
Shear	0.00000
Mosaic	1.00000
Mix up	1.00000
Flip up-down	Horizontal, vertical
Rotation	30°, 45°, 60°, 90°, 180°

**Figure 6:**
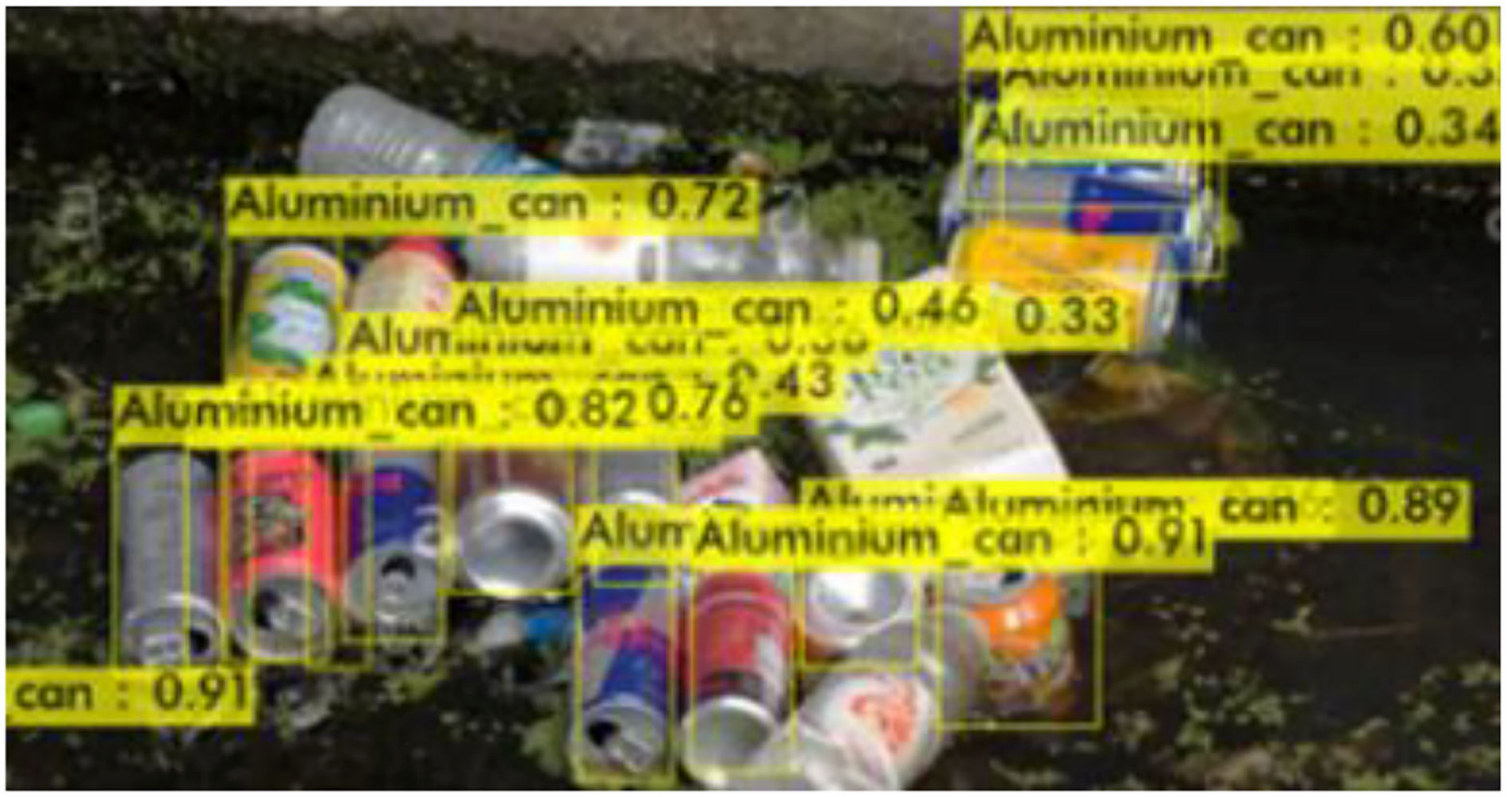
Aluminum cans.

**Figure 7:**
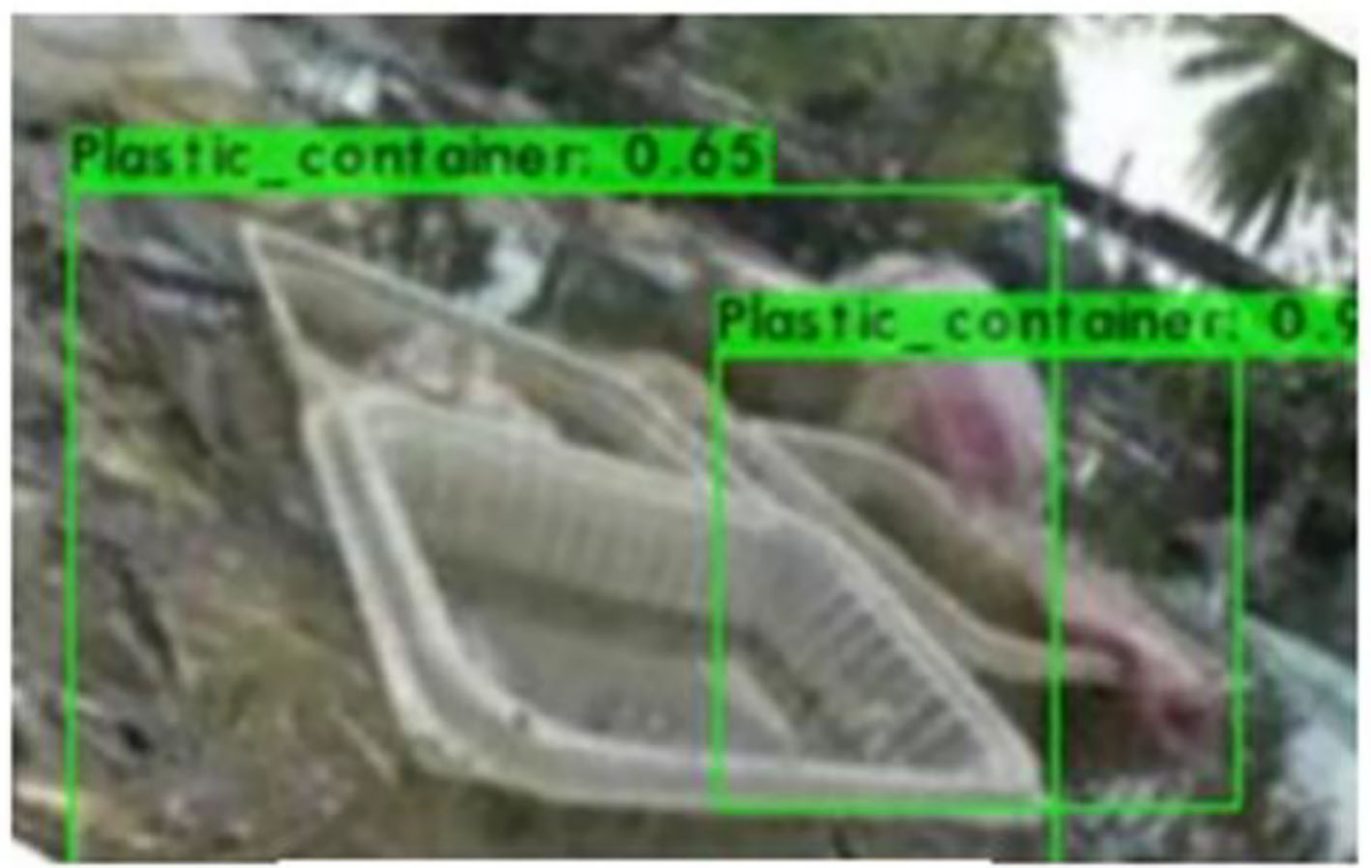
Plastic containers.

**Figure 8:**
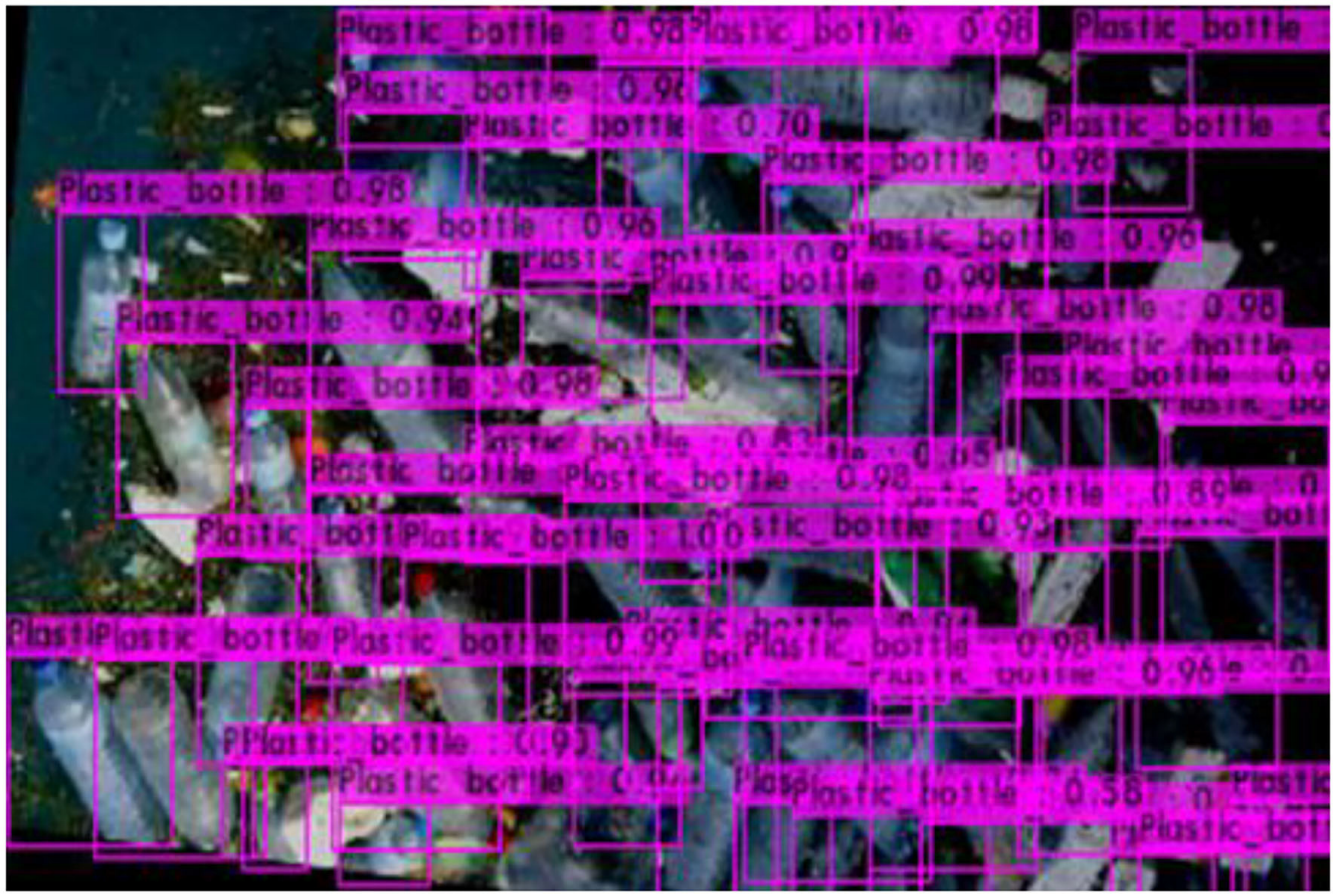
Plastic bottles.

**Figure 9:**
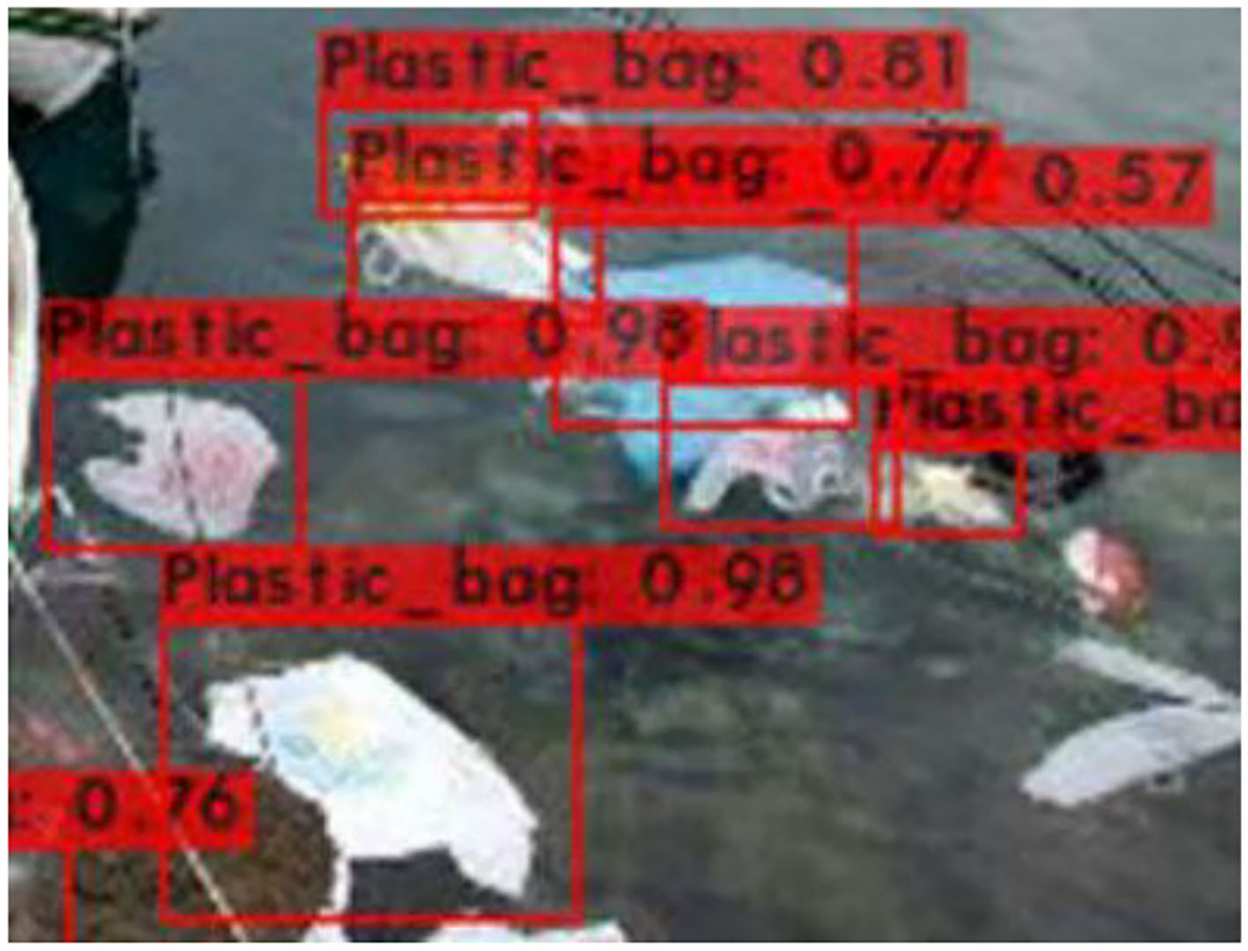
Plastic bags.

Figures 6–9 show several detected objects, such as aluminum cans, plastic containers, plastic bottles, and plastic bags, when using the IoU threshold of 0.3.

**Figure 10:**
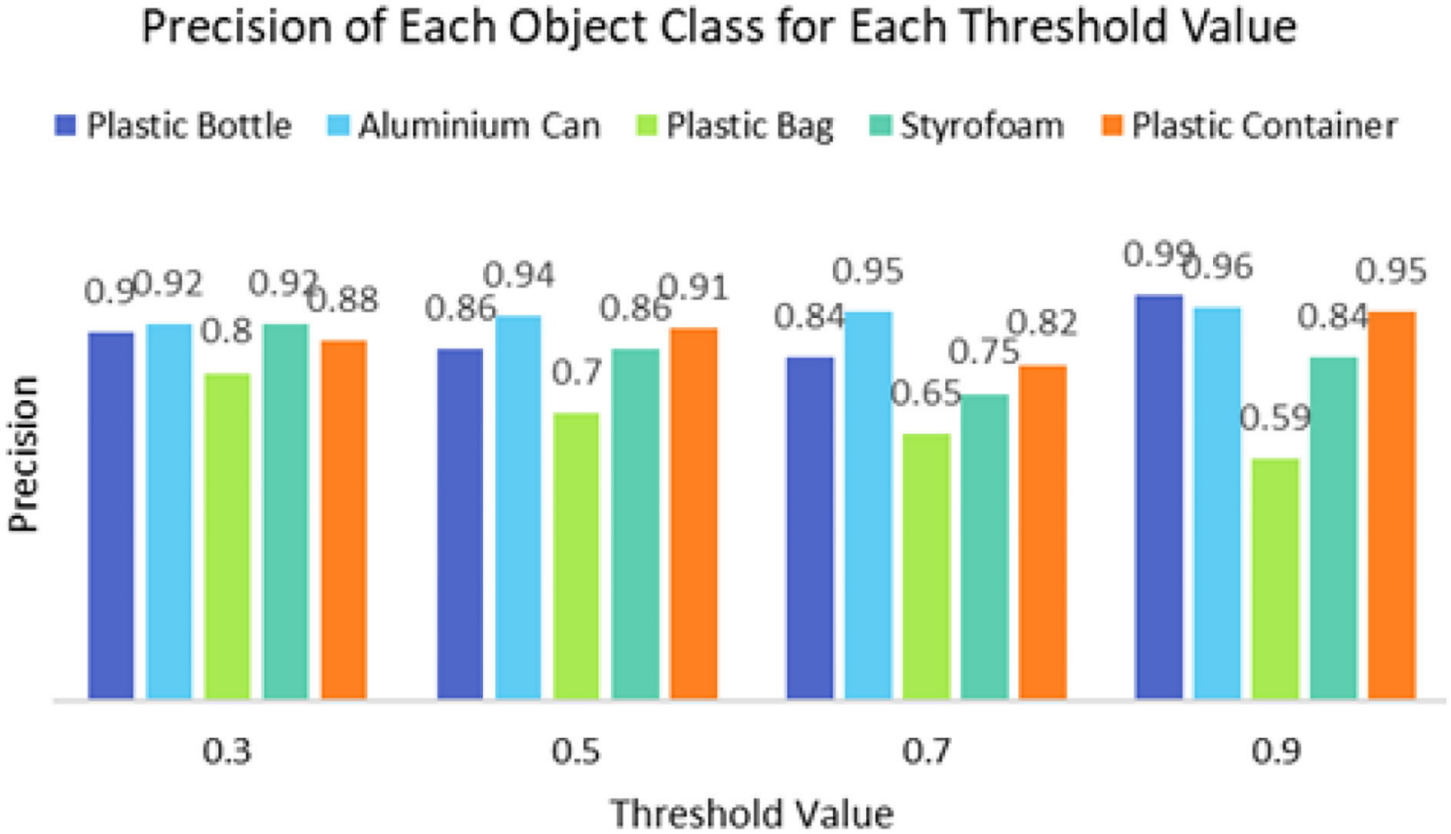
The precision of the object detector using a different threshold value.

**Figure 11:**
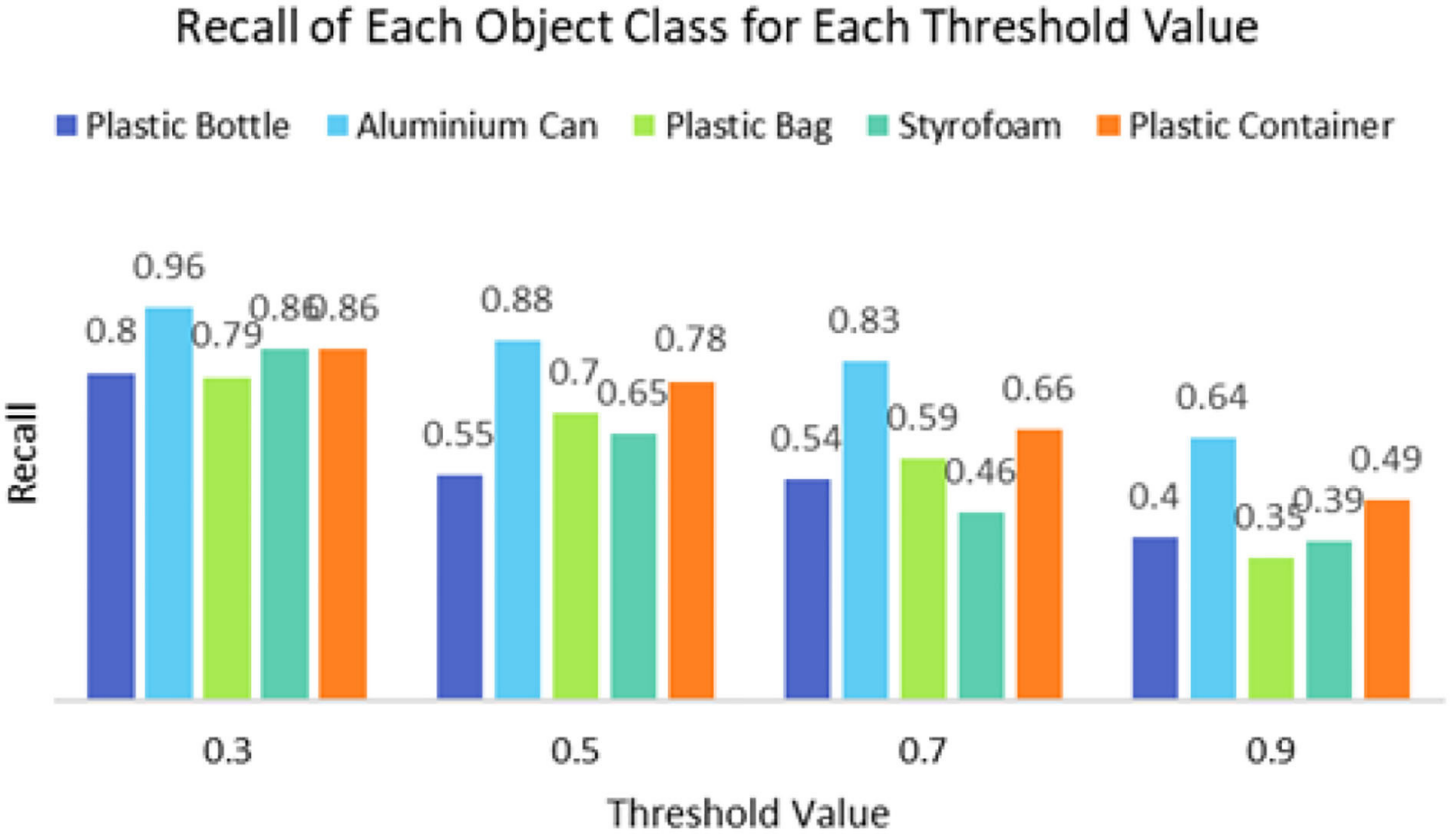
The recall of the object detector using a different threshold value.

**Figure 12:**
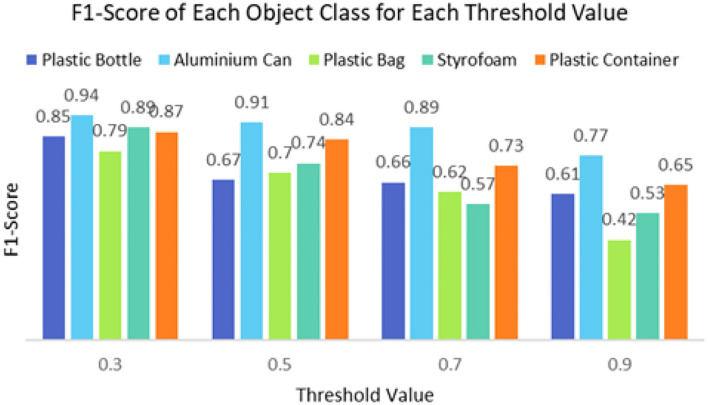
The F1-score of the object detector using different threshold values.

It can be seen that the proposed detection model was able to detect multiple objects in an image even under an occluded condition which justifies its feasibility in detecting floating debris for the river cleaning robot. The plastic bag class has the lowest precision value which indicates that detecting plastic bags is the most challenging task as compared to detecting other types of debris. This is because the plastic bag has complex physical characteristics thus making it challenging to be detected. For analysis purposes, the detection of every image is tested using four threshold values. As shown in Figure 10, different IoU thresholds will produce different detection results because threshold values limit the confidence of the object detector to detect certain objects. Even though the threshold of 0.9 produces the highest precision, it produces the lowest performance in terms of Recall and F1-score values as shown in Figures 11, 12, respectively.

**Figure 13:**
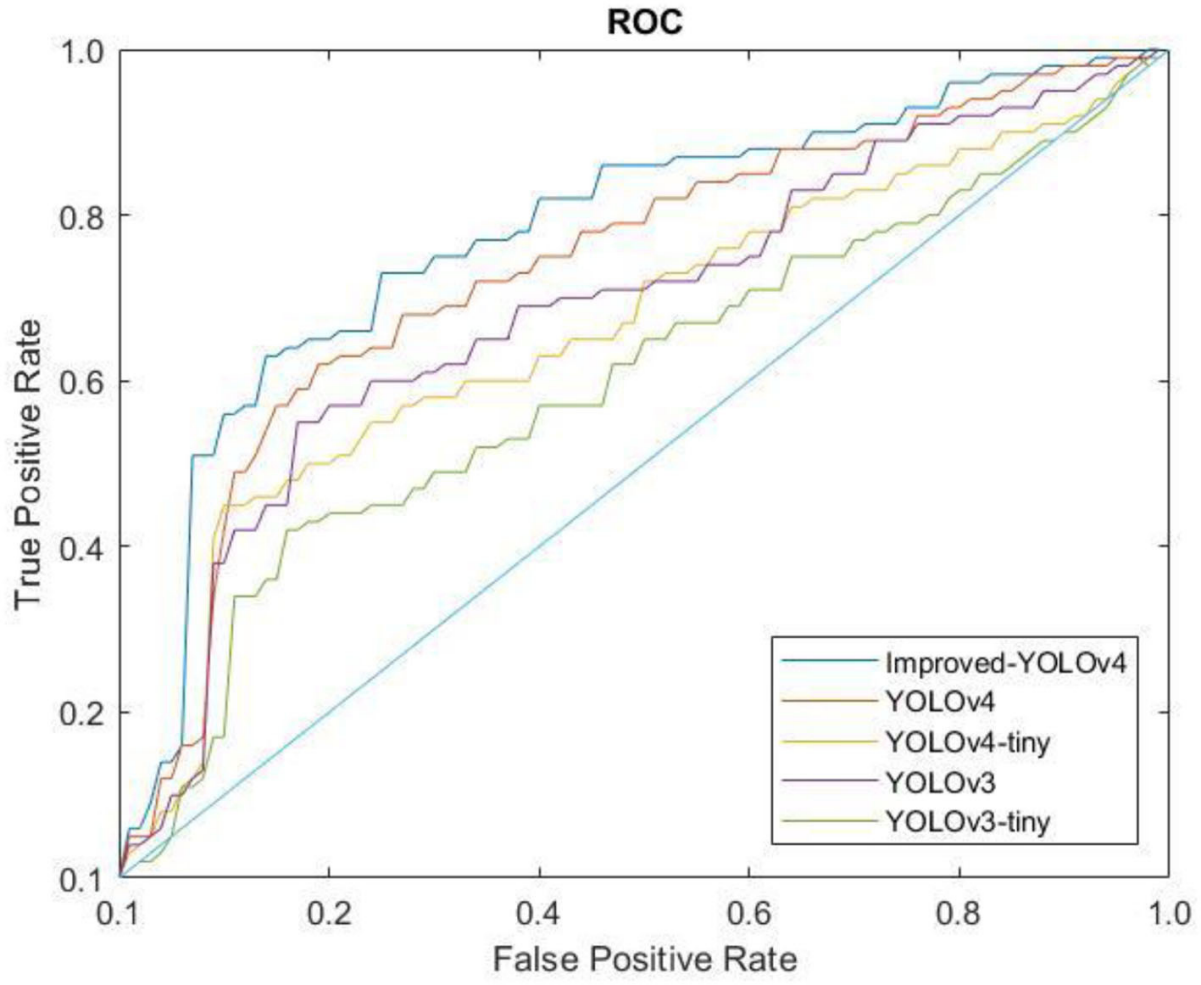
The ROC curves of the proposed model and other models.

A Receiver Operating Characteristic (ROC) curve is computed to measure the performance of the proposed classifier. Figure 13 shows the ROC curves of the proposed model and several YOLO models for comparison purpose to validate the contribution of the optimized model to the conventional YOLO models. Generally, the ROC curve indicates the trade-off between sensitivity and specificity. A high sensitivity value corresponds to high negative predictive output while high specificity corresponds to high positive predictive output. As can be seen, the ROC curve of the proposed model is the highest in the top-left corner, which indicates better classification performance compared to other models. Moreover, the performance of the proposed model is benchmarked with state-of-the-art models as shown in Table 4. It can be seen that the proposed YOLO model provides the highest mAP, F1-score, and Recall values after optimizing the network structure of the conventional YOLOv4 model. Hence, this justifies the contribution of the proposed model in detecting different types of solid waste for the riverine monitoring system. A detection model that has high precision could assist the cleaning robots to complete the cleaning tasks more efficiently. A real-time and high-precision detection technique is crucial to achieving the successful collection of water surface garbage based on machine vision.

**Table 4:** Performance comparison of the proposed model with other YOLO models.

**YOLO models**	**mAP (%)**	**F1-Score**	**Recall (%)**
Yolov3-tiny	52.5	0.4	40
Yolov4-tiny	58.3	0.5	51
Yolov3	65.8	0.6	62
Yolov4	71.2	0.7	68
The proposed work	89.0	0.8	86

In addition, Table 5 benchmarks the proposed detection model with previous works on garbage detection. The performance of the proposed YOLO-v4 model is evaluated on 2,481 test images and has produced the highest mAP value of 89% as compared to the works in Watanabe et al. (12) and Fulton et al. (28). Despite achieving the highest mAP value of 91%, the work in Li et al. (16) only focused on detecting three classes of objects, which are plastic bottles, plastic bags, and styrofoam. Meanwhile, the proposed work focused on detecting five classes of debris (styrofoam, plastic bags, plastic bottles, plastic containers, and aluminum cans). Hence, it can be concluded that the proposed detection model is considered feasible due to its ability to detect more types of debris accurately compared to previous works.

**Table 5:** Benchmarking the proposed work with previous works on garbage detection.

**Work**	**Data**	**mAP**
Watanabe et al. (12) applied YOLO-v3 on 37 test images	4 classes (plastic bottles, plastic bag, drift wood, and other debris)	77.2%
Fulton et al. (28) applied Faster R-CNN on 820 test images	3 classes (plastic debris, biological materials and man-made objects)	81 %
Li et al. (16) applied modified YOLO-v3 on 301 test images	3 classes (plastic bottle, plastic bag, and Styrofoam)	91.4%
The proposed work on 2,481 test images	5 classes (styrofoam, plastic bag, plastic bottle, plastic container, and aluminum can)	89 %

## Conclusion

In conclusion, an automated detection system based on the optimized YOLO model is developed to detect floating solid wastes that include plastic bottles, aluminum cans, plastic bags, styrofoam, and plastic container. In this work, the proposed model optimized the network structure of the conventional YOLOv4 model that includes (i) modification of CSPDark-Net53 into the backbone to overcome limitations due to training time, (ii) adoption of Hard-Swish activation function, and (iii) improved PANet in the Neck module to aid the feature extraction process. The performance of the proposed YOLO model is compared with previous works and has shown promising results with an mAP value of 89%. This research demonstrates that computer vision system plays an important role in environmental monitoring and provides novel insights for improved decision-making and sustainable management. In a nutshell, this study is important for riverine management in urban landscapes since the river is an important part of urban ecological civilization and human health.

## Data availability statement

The raw data supporting the conclusions of this article will be made available by the authors upon request.

## Author contributions

NZ contributed to the development of the idea and participated in all phases. UK conducted the data collection/analysis and manuscript preparation. KH and AM performed the analysis with constructive discussions. MA prepared the figures and manuscript. All authors have read and approved the final manuscript.

## Funding

This research was supported by Industry-Driven Innovation Grant (IDIG) by Universiti Malaya with project number PPSI-2020-CLUSTER-SD01.

## Conflict of interest

The authors declare that the research was conducted in the absence of any commercial or financial relationships that could be construed as a potential conflict of interest.

## Publisher's note

All claims expressed in this article are solely those of the authors and do not necessarily represent those of their affiliated organizations, or those of the publisher, the editors and the reviewers. Any product that may be evaluated in this article, or claim that may be made by its manufacturer, is not guaranteed or endorsed by the publisher.
